# Outcomes and Techniques of Robotic-Assisted Partial Nephrectomy (RAPN) for Renal Hilar Masses: A Comprehensive Systematic Review

**DOI:** 10.3390/cancers16040693

**Published:** 2024-02-06

**Authors:** Savio Domenico Pandolfo, Zhenjie Wu, Riccardo Campi, Riccardo Bertolo, Daniele Amparore, Andrea Mari, Paolo Verze, Celeste Manfredi, Antonio Franco, Francesco Ditonno, Clara Cerrato, Matteo Ferro, Francesco Lasorsa, Roberto Contieri, Luigi Napolitano, Antonio Tufano, Giuseppe Lucarelli, Simone Cilio, Sisto Perdonà, Salvatore Siracusano, Riccardo Autorino, Achille Aveta

**Affiliations:** 1 Department of Urology, University of L’Aquila, 67010 L’Aquila, Italy; salvatore.siracusano@univaq.it; 2 Department of Neurosciences and Reproductive Sciences and Odontostomatology, University of Naples “Federico II”, 80131 Naples, Italy; luiginap89@gmail.com (L.N.); simocilio.av@gmail.com (S.C.); achille-aveta@hotmail.it (A.A.); 3 Department of Medicine and Surgery, Scuola Medica Salernitana, University of Salerno, 84081 Fisciano, Italy; pverze@gmail.com; 4 Department of Urology, Changhai Hospital, Naval Medical University, Shanghai 200433, China; wuzhenjie17@163.com; 5 Urological Robotic Surgery and Renal Transplantation Unit, Careggi Hospital, University of Florence, 50121 Firenze, Italy; riccardo.campi@gmail.com (R.C.); andreamari08@gmail.com (A.M.); 6 Department of Urology, University of Verona, 37100 Verona, Italy; riccardobertolo@hotmail.it (R.B.); francesco.ditonno@icloud.com (F.D.); 7 Division of Urology, Department of Oncology, School of Medicine, University of Turin, San Luigi Hospital, Orbassano, 10043 Turin, Italy; danieleamparore@hotmail.it; 8 Department of Urology, Rush University Medical Center, Chicago, IL 60637, USA; manfredi.celeste@gmail.com (C.M.); anto.franco@hotmail.it (A.F.); ricautor@gmail.com (R.A.); 9 Unit of Urology, Department of Woman, Child and General and Specialized Surgery, University of Campania “Luigi Vanvitelli”, 80131 Naples, Italy; 10 Department of Urology, Sant’Andrea Hospital, La Sapienza University, 00189 Rome, Italy; 11 Urology Unit, University Hospital Southampton NHS Trust, Southampton SO16 6YD, UK; clara.cerrato01@gmail.com; 12 Division of Urology, IRCCS—European Institute of Oncology, 71013 Milan, Italy; drmatteoferro@gmail.com; 13 Department of Precision and Regenerative Medicine and Ionian Area-Urology, Andrology and Kidney Transplantation Unit, University of Bari “Aldo Moro”, 70124 Bari, Italy; francesco-lasorsa96@libero.it (F.L.); giuseppe.lucarelli@inwind.it (G.L.); 14 Department of Biomedical Sciences, Humanitas University, 20072 Pieve Emanuele, Italy; contieri.ro@gmail.com; 15 Department of Urology, Istituto Nazionale Tumori, IRCCS, “Fondazione G. Pascale”, 80131 Naples, Italy; antonio.tufano91@gmail.com (A.T.); s.perdona@istitutotumori.na.it (S.P.)

**Keywords:** complex renal mass, hilar renal mass, hilum, renorrhaphy, robot-assisted partial nephrectomy, enucleation

## Abstract

**Simple Summary:**

This study provides a thorough review of robot-assisted partial nephrectomy (RAPN) in managing renal hilar masses. It focuses on the evaluation of RAPN’s effectiveness and the exploration of varied surgical methods for these complex tumors. The research underscores the reliability of RAPN and emerging surgical techniques in addressing these challenges with a manageable risk of complications. The findings are poised to significantly contribute to the medical community’s understanding and management of renal hilar masses, especially in terms of effectively balancing treatment efficacy and complication risks.

**Abstract:**

**Background**: Robot-assisted partial nephrectomy (RAPN) is increasingly being employed in the management of renal cell carcinoma (RCC) and it is expanding in the field of complex renal tumors. The aim of this systematic review was to consolidate and assess the results of RAPN when dealing with entirely central hilar masses and to examine the various methods used to address the surgical difficulties associated with them. **Methods**: A thorough literature search in September 2023 across various databases focused on RAPN for renal hilar masses, adhering to PRISMA guidelines. The primary goal was to evaluate RAPN’s surgical and functional outcomes, with a secondary aim of examining different surgical techniques. Out of 1250 records, 13 full-text manuscripts were reviewed. **Results**: Evidence is growing in favor of RAPN for renal hilar masses. Despite a predominance of retrospective studies and a lack of long-term data, RAPN shows positive surgical outcomes and preserves renal function without compromising cancer treatment effectiveness. Innovative suturing and clamping methods are emerging in surgical management. **Conclusions**: RAPN is a promising technique for managing renal hilar masses in RCC, offering effective surgical outcomes and renal function preservation. The study highlights the need for more long-term data and prospective studies to further validate these findings.

## 1. Introduction

Renal cell carcinoma (RCC) is a relatively common disease, with approximately 79,000 new cases and 14,000 deaths estimated in the US during 2022 [1,2,3]. Nowadays, alternatives to surgery are available, especially in the realm of ablative therapies [4]. Recent multicentric reports have proven their effectiveness even in complex surgical settings such as solitary kidney [5] and completely endolithic renal masses [6]. Although the growing evidence tends toward alternatives to surgery, current European Association of Urology (EAU) Guidelines recommend offering partial nephrectomy (PN) in patients with T1 tumors and in selected cases of T2 cancers (e.g., solitary kidney or chronic kidney disease [CKD]), if technically feasible [7,8]. Furthermore, they do not recommend a specific surgical approach to perform PN (open [OPN], laparoscopic [LPN], robotic [RAPN]) [9,10,11,12].

However, it is widely accepted that RAPN offers enhanced dexterity and improved visualization. The high-resolution, three-dimensional visualization offered by robotic systems significantly improves a surgeon’s ability to expand the indications for nephron-sparing surgery (NSS) to larger, deeper, and more complex renal tumors [13]. This array of benefits has contributed significantly to the widespread adoption of robotic surgery, especially in complex cases. The ability of robotic systems to navigate and operate in confined or challenging anatomical spaces with high precision has made them a preferred choice for managing complex RCC cases [14]. As such, while there is no definitive evidence asserting the superiority of robotic techniques over traditional methods, their perceived benefits in terms of surgical accuracy have led to their diffusion.

In this context, kidney tumor location remains a fundamental parameter in determining whether a PN is a viable option [15,16]. More specifically, hilar location is as a key feature defining tumor complexity [17,18,19]. Indeed, hilar masses represent intricate tumors, often characterized by their proximity to the main renal artery or vein, and/or the renal pelvis on preoperative computed tomography scans [20,21]. These situations present significant challenges for the urologist [22,23]. Consequently, hilar lesions place the patient at greater surgical risk when a PN is offered [17,24].

The aim of this study was to analyze the current evidence on RAPN for the management of renal hilar masses.

## 2. Materials and Methods

In September 2023, a systematic review of the available literature was conducted by searching through databases such as MEDLINE (PubMed), Scopus, Web of Science Core Collection, and the Cochrane Library. Preferred Reporting Items for Systematic Review and Meta-Analysis (PRISMA) recommendations were followed to report the data [25,26]. The protocol was registered in the International Prospective Register of Systematic Review (PROSPERO) with ID CRD42023456397 [27].

Different combinations of the following keywords were used to search for articles by all fields: robot, robotic, partial, kidney, renal, nephrectomy, nephron, sparing, hilar, hilum, complex, technology, imaging, ultrasound, ultrasonography, 3D, three-dimensional, hologram. Search results were filtered by language (English only), species (human), publication type (article), and year (2010–2023).

Study eligibility was defined using the PICOS (patient, intervention, comparator, outcome, study type) approach [28,29]:

(P) adults (>18 years) with a diagnosis of RCC;
(I) RAPN;
(C) comparison between different surgical techniques present or absent;
(O) at least one of the following: surgical outcomes [operative time (OT), estimated blood loss (EBL), warm ischemia time (WIT), intraoperative complications, major postoperative complications (Clavien–Dindo ≥ III) [30], conversion rate], functional outcomes [eGFR preservation (%), upstaging CKD, Trifecta achievement [31]]; oncologic outcomes [positive surgical margin (PSM), recurrence rate, metastases rate];
(S) prospective or retrospective design.

The initial screening involved reviewing the titles and abstracts of papers to assess their eligibility for inclusion. Afterwards, a more comprehensive assessment was performed on the full-text articles. Further relevant studies were identified by examining the reference lists of the selected articles. Conference abstracts, case reports, small case series (fewer than 10 cases), reviews, letters to the editor, and editorial comments were excluded from consideration.

The following items were recorded for each eligible paper: first author, publication year, study design, number of patients, age of patients, surgical procedure, tumor size, R.E.N.A.L. score, OT, EBL, WIT, PSM, follow-up, eGFR change, complications, recurrence rate, and metastases rate. This comprehensive data collection allowed for a detailed analysis and comparison across studies, contributing to a robust synthesis of findings.

Study screening, article selection, data extraction, and risk of bias assessment were performed independently by two authors (SDP, AA), while a third senior author (CM) supervised and resolved disagreement. This collaborative approach ensured a rigorous and transparent review process, minimizing potential biases and enhancing the credibility of the findings.

## 3. Results

### 3.1. Study Identification and Selection

**Figure 1:**
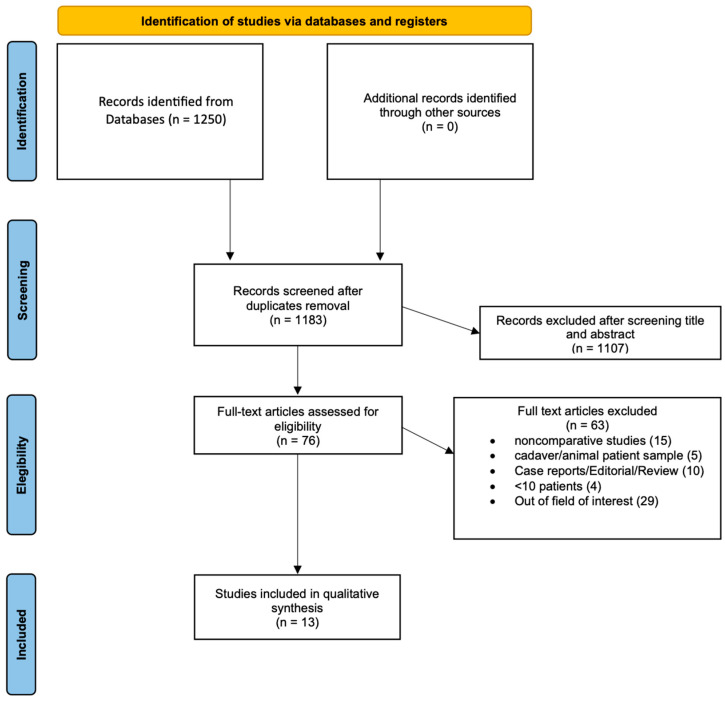
PRISMA 2020 flow diagram for new systematic reviewers which included searchers of databases and registers only. For more information, visit http://www.prisma-statement.org/ (accessed on 15 September 2023).

The PRISMA flow chart of the study selection process is shown in Figure 1. The initial search identified 1250 studies from 2010 to June 2023. Of these, 67 were excluded for duplication. After applying eligibility criteria, another 1148 records were excluded. Finally, a total of 13 studies evaluating 4871 patients were included [32,33,34,35,36,37,38,39,40,41,42,43,44].

### 3.2. Study Characteristics and Methodological Quality Assessment

All studies had a retrospective design except for Hinata et al. who reported prospective data [39]. Five studies, including 2941 subjects, compared outcomes between hilar and non-hilar tumors [32,33,34,35,44]; one paper, assessing 31 patients, compared RAPN and OPN [37]; two articles, evaluating 178 subjects, compared RAPN with LPN [38,39]; one document from the French National Network of Research on Kidney Cancer compared the type of hilar control approach (off-clamp vs. on-clamp) in 1359 patients [36]. Finally, one article analyzed and compared five patients who underwent RAPN using a novel renorrhaphy technique, V-hilar suture (VHS), with 10 patients who underwent standard RAPN [43].

The risk of bias (RoB) of selected studies was evaluated with the Newcastle-Ottawa Scale (NOS) [45]. Arbitrarily, a total NOS score of 0–5 was associated with a low quality, 6 with an intermediate quality, and 7–9 with a high quality. The level of evidence (LoE) was described following the directions of the Oxford Centre for Evidence-Based Medicine 2011 (range: 1–5) [46]. The LoE of all studies was 4. The NOS ranged between 7 and 8 points among the articles.

### 3.3. Patient and Tumor Characteristics

The main characteristics of the included studies are reported in Table 1.

Mean tumor size varied from 2.6 to 5.0 cm, while R.E.N.A.L. nephrometry ranged between 7 and 10. The OT varied widely with a range from 85 to 293.6 min where the minimum OT moved to 144.1 without considering the off-clamp cohort by Ferriero et al. [42]. Specifically, when considering LPN, the OT ranged from 126 to 146 min, while Miyake et al. reported a mean of 203.7 min. for the OPN cohort [37]. The age range spans from a minimum of 50.4 years to a maximum of 64.4 years. The youngest cohort was reported by Tyagi and colleagues in the hilar arm [32] while the oldest was described in the OPN group in Miyake et al.’s report [37].

### 3.4. Surgical Outcomes

The surgical outcomes of the included studies are reported in Table 1.

A wide range of EBL was reported (86–653 mL). Warm ischemia was applied in all studies except for the off-clamp arm in Mellouki et al. and in the article by Ferriero et al. [36,42]. WIT ranged between 15 and 39.9 min. Precisely, various clamping techniques such as selective, super selective, and early unclamping were allowed by surgeons’ preferences and reported in the on-clamp groups by Mellouki et al., Zhang et al., and Hinata et al. [36,39,41].

Intraoperative complication rates were reported only by Chen et al. and were comparable between LPN and RAPN (2 vs. 4; *p* = 0.56) [38]. Chen et al. in the LPN group and Lu et al. in their hilar cohort both observed a single patient undergoing conversion to an open procedure [35,38]. Sunaryo et al. noted a significant shift towards radicalization in the hilar cohort, whereas Chen et al. only reported a higher trend in the LPN group without reaching statistical significance [33,38]. Major postoperative complications were described in 10 series [32,33,35,36,38,40,41,42,43,44] with a percentage variable between 0 and 6.3% excluding preliminary outcomes reported by Khalifeh and colleagues [43].

### 3.5. Functional Outcomes

Seven studies reported functional outcomes with the eGFR drop floating from 0 to −16.8 mL/min [33,35,37,38,39,42,43]. Lu et al. reported an eGFR decrease of 4.6 and 6.4 mL/min for hilar and non-hilar cohorts, respectively. Instead, Sunaryo and colleagues reported the worst functional outcomes, with a significant drop in eGFR of −16.8 mL/min, for the hilar group and 12.6 mL/min for the non-hilar group, indicating a substantial decline in kidney function in both cohorts [33].

Finally, only Tyagi et al. reported pentafecta achievement with values of 34.1% and 58.5% for hilar vs. non-hilar, respectively [32].

### 3.6. Oncological Outcomes

Eight studies reported follow-up data [32,34,35,38,39,40,42,43], and two recorded very short follow-ups (3 and 7.4 months) [34,43]; the maximum follow-up was reported by Gao et al. (48 months) [40]. Seven studies reported functional outcomes with the eGFR drop floating from 0 to −16.8 mL/min [35,37,38,39,40,42,43].

PSM was the most reported oncologic outcome [32,33,34,35,36,37,38,40,41,42,44], varying from 0 to a maximum of 10.7% reported by Mellouki et al. [36]. Four studies reported a recurrence rate, ranging between 0 and 5% [36,37,40,42]. Only Mellouki et al. described metastases rate with a higher trend for the off-clamp arm [36].

## 4. Discussion

Hilar tumors present several challenges to minimally invasive surgery, because of their close proximity to the blood vessels and collecting system, as well as the absence of a parenchymal margin near the renal hilum for renorrhaphy [47,48,49]. Hence, these have been traditionally treated via open partial nephrectomy (OPN) [50,51,52,53]. In 2005, Gill et al. reported its feasibility with LPN [54]. RAPN has been considered an implementation of LPN, capable of overcoming the technical challenges associated with the latter. Several studies demonstrated that RAPN is more favorable than LPN in challenging cases [55,56,57]. Miyake and colleagues documented that RAPN appears to yield more favorable results in terms of morbidity when compared to OPN, while maintaining an equivalent level of oncological effectiveness and safety. [37]. Chen and Hinata found LPN to have higher WIT but similar PSM rate when compared to RAPN in a single center and multi-institutional setting [38,39].

### 4.1. Comparison between Hilar vs. Non-Hilar Tumors

When looking at tumor features, hilar masses were consistently larger in size compared to non-hilar masses [58,59]. One of the first reports is from Dulabon et al. with a multi-institutive analysis [44]. Longer WIT for the hilar cohort (26.3 vs. 19.6 min) was the main difference. Afterward, Eyraud et al. reported a single-center experience comparing 70 hilar vs. 297 non-hilar tumors [34]. Greater size, higher complexity, and a more meticulous vascular dissection may explain the longer OT (210 min versus 180 min) and WIT (27 min vs. 17 min) in the hilar arm. In their multivariate analysis, the authors observed that the location and size of the masses were recognized as independent predictors of WIT. This observation aligns with the findings of Ficarra et al., where anatomic tumor characteristics as determined by the PADUA classification score were independent predictors of WIT [60]. Longer WIT were also reported by Cacciamani et al. in a meta-analysis for the hilar tumors compared to non-hilar [61].

Later, two single-center and one multi-institutional report were published [32,33,35]. Higher EBL was observed in cases of hilar tumors in the reports by Lu et al. and in Tyagi et al., while no difference was found in the matched cohorts in Sunaryo and colleagues’ experience. While statistically significant in the majority of the studies, the rise in EBL was unlikely to have clinical significance, given that the transfusion rates for both hilar and non-hilar tumors were similar.

Functional outcomes in such challenging scenarios have always been a concern [62,63,64,65]. Regarding the studies included in the analysis, Tyagi at al. did not identify disparities in terms of eGFR, CKD upstaging, or the preservation of 90% eGFR at the one-year mark. [32]. Lu et al. did not observe any noteworthy distinction in eGFR values after 6 and 12 months [35]. Similarly, Eyraud et al. did not report any variance in terms of CKD upstaging or alterations in eGFR [34]. Sunaryo et al. in a multivariate analysis reported worse eGFR decline over time and no differences in eGFR at discharge [33]. Despite the higher complexity stemming from the anatomical location associated with longer WIT and EBL, all the studies concur that there is an equivalent restoration of renal function when compared to the non-hilar cases. This implies that when a successful RAPN is carried out, the level of complexity associated with the procedure does not seem to have an impact on the ultimate functional outcome.

### 4.2. Clamping Techniques for Renal Hilar Masses

The primary objective of PN is to achieve complete removal of the tumor with clear surgical boundaries, while also minimizing global WIT and optimizing preoperative eGFR [66,67,68]. These three factors have been shown to be important predictors of postoperative renal function following RAPN [14,20]. Moreover, recent tools have been developed to predict renal function outcomes post-surgery in order to improve patient management and guide decision-making in RCC-challenging settings [69,70]. Focusing on hilar masses, all of this becomes even more complex due to their proximity to the main renal artery or vein, and/or the renal pelvis [71,72,73,74,75]. For this reason, the optimal management of the renal hilum in these cases is still ongoing [76,77,78]. Two reports focused on the off-clamp approach. Ferriero et al. reported a small-size experience in their tertiary referral center with excellent perioperative, functional, and oncologic outcomes in their series. Ten off-clamp RAPN for purely hilar masses were performed, and a blunt dissection from the collecting system and hilar vessels were employed for the tumor excision; no sutures were performed to the resection bed to prevent damage to the collecting system or blood vessels [42]. Moreover, Mellouki et al. focused on the comparison between on-clamp vs. off-clamp approaches for hilar masses especially looking at oncological outcomes. Although the authors believed that the on-clamp approach could offer advantages in terms of better visualization during the resection, no significant difference in terms of PSM rate were found between the two groups (5.6% vs. 11%; *p* = 0.1) [36]. Authors reported retrospective data from operative reports without any preoperative intention of the renal hilum clamping strategy. Recent data from RCT CLOCK and CLOCK II reported how the decision to switch from the on-clamp to off-clamp approach may be influenced by factors related to tumor masses and their complexity [79].

### 4.3. Resection Techniques for Renal Hilar Masses

In the early practice of PN, it was recommended to resect at least a 1-cm margin of healthy tissue around the tumor’s pseudocapsule [80,81]. However, there has been a notable evolution from the traditional resection technique towards the more anatomical approach of tumor enucleation [82]. Moreover, some experts suggest that tumor enucleation, which prioritizes retaining more kidney tissue without compromising cancer control, could offer distinct advantages over the conventional method of PN [83,84]. This evolution in technique reflects a deeper understanding of the importance of conserving functional renal tissue, potentially leading to better postoperative outcomes without compromising the effectiveness of cancer treatment [85].

In the current literature, there is a recognition of the unique challenges presented by renal hilar masses, primarily due to their close proximity to blood vessels and the collecting system, coupled with the absence or scarcity of a parenchymal margin near the renal hilum [20,47]. Furthermore, these tumors are recognized in certain studies as a risk factor for the progression of the tumor stage from T1 to T3a, primarily because of this closeness to the renal sinus fat and vasculature [86,87]. In this point of view, the identification of an enucleative plane, combined with the use of advanced surgical techniques such as robot-assisted surgery, can significantly enhance precision during the procedure with adequate surgeon’s skill and experience [88]. The combination of these surgical techniques with the expertise and judgment of experienced surgeons forms a robust approach to effectively manage these complex cases, reducing the likelihood of PSM and potentially improving oncological outcomes [89].

Consequently, this consensus extends to the preferred surgical approach for these masses, with a strong endorsement for enucleative techniques which is the most common reported approach for managing a hilar lesion, adhering closely to the lesion while respecting the limits of the pseudocapsule [21,49]. This strategic choice in surgical method demonstrates an evolved understanding of renal anatomy and pathology, prioritizing organ preservation and functionality alongside effective cancer management [20,90,91].

### 4.4. Suturing Techniques for Renal Hilar Masses

Renorrhaphy stands out as one of the key steps during RAPN, and this becomes technically more demanding in case of hilar masses due to its close proximity to critical structures [92,93,94,95]. Several strategies have been adopted and described to date [96,97,98,99,100,101].

Early reports have mentioned surgical bolster to fill in the defect [102,103]. A newer renorrhaphy technique, which involves the use of sliding clips instead of knots, has offered quicker renal suturing, a reduced learning curve, and a decrease in the “cheese-cutting effect” [104]. The common contemporary renorrhaphy technique typically consists of two tiers of suturing: a deeper layer at the excision site and a shallower layer [96,101,105,106,107,108,109,110]. However, in the case of larger defects, achieving complete reapproximation of the capsule may be challenging, particularly when dealing with hilar renal masses to prevent compression of the renal hilum [111,112,113].

In this context, Gao et al. and Khalifeh et al. proposed two novel tension-free renorrhaphy techniques in response to the tension in suturing extensive wounds and its potential risk of “cheese-cutting” strength [40,43]. Khalifeh et al. reported preliminary outcomes of a novel renorrhaphy technique for managing hilar masses, the “V-hilar suture” (VHS) [43]. The authors suggest a method that involves the utilization of an 8-inch 2-0 Vicryl suture, which has been pre-equipped with a knot and Hem-O-Lok clip at its free end. This suture is employed for an inner-layer suture, extending from one vertex to the opposite apex of the renal parenchyma. At the termination of the suture line, it is passed through the capsule and secured using two sliding Hem-O-Lok clips. The inner-layer sutures are adapted to reshape the renal parenchyma by medially repositioning the central-lateral boundary of the resection bed. The technique aims to provide hemostatic management of larger parenchymal blood vessels, sealing off access points to the collecting system, and restructuring the hilum into a V-shaped configuration [43]. The authors reported preliminary outcomes for a median nephrometry R.E.N.A.L. score of 10, indicating the high complexity of these cases. Thus, key perioperative factors like OT, EBL, WIT, and the eGFR reduction tended to be higher compared to the general population undergoing RARP at the same institution. The “Garland technique” was described by Gao and colleagues, demonstrating its feasibility and comparable outcomes to the standard. To be more specific, the authors described an uninterrupted suture that covers all layers of tissue, employing a barbed thread (0-QUILL). This suture begins in a retrograde manner from the hilum towards the parenchyma. Each stitch follows the contours of major blood vessels and is fastened with clips on the renal capsule, creating a closure that resembles a garland [40].

### 4.5. Complications and Reliability

Despite the heterogeneity of the included comparative studies, major postoperative complications—whether examining non-hilar versus hilar tumors, off-clamp versus on-clamp techniques, or laparoscopic LPN vs. RAPN—did not show a significantly different outcome in terms of complication rates. Lu et al., despite larger tumor size and higher rate of collecting system entry compared with the non-hilar group, reported no significant increase in overall and major complication rates [35]. Tyagi et al. found that hilar location was associated with poorer trifecta outcomes compared to non-hilar tumors. However, hilar location per se was not found as an independent predictor of overall complications and trifecta and pentafecta outcomes [32]. With regards to vascular hilum management, Mellouki and colleagues reported Clavien–Dindo grade ≥ III marginally higher in the off-clamp group (6.2%) versus the on-clamp group (5.7%), with no significant difference (*p* = 0.5) [36]. Moreover, recent data from the prospective randomized Trial—CLOCK—showed that the transition from off- to on-clamp management can be addressed in daily practice, and it is related to renal mass diameter and complexity. However, this shift did not translate into significant differences in postoperative complications or renal function at 6 months [79,114].

An encouraging trend has also been observed in reports that describe novel techniques. Gao et al. reported Clavien I e II complications in 7.0% of patients, indicating a favorable outcome with the described “Garland” surgical technique [40]. Khalifeh and colleagues reported preliminary outcomes for the novel VHS technique, which was specifically tailored to achieve collecting systems and parenchymal closure, and no major postoperative complication where noted [43].

The consistency of these results across different studies reinforces the notion that despite the complexity and challenges associated with hilar masses, current RAPN and emerging surgical techniques are capable of effectively managing these conditions with a manageable risk of complications.

### 4.6. Strengths and Limitations

The review presents a detailed analysis of various outcomes, including surgical, functional, and oncologic parameters. The comparisons among different surgical techniques, such as robotic, laparoscopic, and open approaches, provide a comprehensive view of the available evidence.

The review acknowledges the limitations of the included studies which are summarized in Supplementary Table S1. A common limitation was the retrospective design, which was present in almost all studies, potentially introducing selection bias and limiting the ability to establish causality. Specifically, of the 13 studies analyzed, 12 have a retrospective design, with two based on multicentric cohorts and another two on national-level analyses. Only one report features a multicentric and prospective design. This significantly limits the robustness of evidence that can be derived from the systematic review of current literature, while simultaneously emphasizing the critical need for well-focused future studies, thereby highlighting the significance of this research scenario.

Furthermore, a lack of comprehensive functional and recurrence outcome data was noted in several studies, which could impede a full evaluation of the effectiveness and safety of the surgical techniques discussed. These collective limitations underline the need for caution in interpreting the review’s findings and highlight the importance of future prospective studies with larger, more diverse cohorts and comprehensive outcome measures to provide more definitive conclusions. In addition, ensuring that patients are aware of the evidence quality and the potential implications this might have on the reliability of the findings is a critical component of informed consent. This transparency helps patients understand the context in which the evidence supporting their treatment options was gathered and allows them to make more informed decisions about their care.

## 5. Conclusions

Over the past decade, substantial evidence has emerged supporting the use of RAPN in managing hilar renal tumors. This SR, predominantly from retrospective studies, has further corroborated that RAPN not only maintains renal functionality effectively with a manageable risk of complications but also ensures oncological safety, a critical consideration in such complex surgical scenarios. Particularly noteworthy are the innovative suturing and clamping techniques that have evolved, broadening the surgical options available and offering alternatives to the landscape of surgical management for these challenging cases.

However, it is crucial to emphasize that a personalized approach is key to optimizing surgical outcomes in those complex surgeries. Indeed, the nuanced decision-making process, tailored to each individual case, should always consider tumor characteristics and the surgeon’s expertise and experience.

In summary, while RAPN emerges as a promising method for treating hilar renal tumors, its effectiveness hinges on a comprehensive, patient-centric approach that balances technical innovation with personalized care.

## Figures and Tables

**Table 1:** RAPN for hilar renal masses: an overview of reported series.

Reference	Study Design	Procedure (*n*)	Size to CT, cm	R.E.N.A.L. Score	OT, min	EBL, mL	WIT, min	PSM, *n*, (%)	Follow-Up, Months	Latest eGFR Change	Major Postop. Complication ^a^, *n* (%)	Recurrence, *n* (%)
Dulabon et al., 2010 [44]	RMC	- RAPN (446) Hilar (41) Nonhilar (405)	3.46 ^a^ 2.88 ^a^	NR	194.5 ^a^ 187.4 ^a^	262.2 ^a^ 208.2 ^a^	26.3 ^a^ 19.6 ^a^	1 (2.4) 6 (1.4)	NR	NR	0 (0) 7 (1.7)	NR
Khalifeh et al., 2012 [43]	RSC	- Novel (5) - Standard (10)	5.02 ^b^ 4.66 ^b^	10 ^b^ 10 ^b^	215.2 ^b^ 195 ^b^	250 ^b^ 575 ^b^	31.6 ^b^ 30.7 ^b^	NR	3 3	−11.4 ^b^ −7.26 ^b^	0 (0) 2 (10)	NR
Eyraud et al., 2013 [34]	RSC	- RAPN (364) Hilar (70) Nohilar (294)	3.9 ^b^ 2.6 ^b^	NR	210 ^b^ 180 ^b^	250 ^b^ 200 ^b^	27 ^b^ 17 ^b^	1 (1.4) 9 (3)	7.4 ^b^ 7.4 ^b^	NR	NR	NR
Miyake et al., 2014 [37]	RSC	- RAPN (16) OPN (15)	3.0 ^a^ 3.2 ^a^	NR	263.0 ^a^ 203.7 ^a^	57.5 ^a^ 653.6 ^a^	23.0 ^a^ 20.3 ^a^	0 0	NR	−10.0 ^a^ −10.4 ^a^	NR	0 (0) 0 (0)
Lu et al., 2018 [35]	RSC	- RAPN (200) Hilar (30) Nonhilar (170)	4.8 ^a^ 3.7 ^a^	9 ^a^ 7.4 ^a^	293.6 ^a^ 240.5 ^a^	418.7 ^a^ 205.8 ^a^	39.9 ^a^ 21.8 ^a^	1 (3.3) 0 (0)	28 ^a^ 32.3 ^a^	−4.6 ^a^ −6.4 ^a^	0 (0) 4 (2.3)	NR
Gao et al., 2020 [40]	RSC	- RAPN (286)	2.6 ^a^	8.2 ^a^	120 ^b^	100 ^b^	18.2 ^b^	3 (1)	48 ^b^	NR	18 (6.3)	3 (1)
Sunaryo et al., 2020 [33]	RMC	- RAPN (1730) Hilar (263) Nonhilar (1467)	3.7 ^b^ 3 ^b^	9 ^b^ 7 ^b^	186 ^b^ 161 ^b^	100 ^a^ 100 ^a^	18 ^b^ 15 ^b^	9 (3.4) 68 (4.6)	NR	−16.8 ^b^ −12.6 ^b^	12 (4.5) 54 (3.7)	NR
Mellouki et al., 2020 [36]	ND	- RAPN (1359) Off-Clamp (224) On-Clamp (1135)	3.6 ^a^ 3.8 ^a^	7 ^a^ 7 ^a^	NR	198.9 ^a^ 229.5 ^a^	17.6 ^a^ NR	12 (5.3) 122 (10.7)	NR	NR	14 (6.2) 65 (5.7)	6 (2.6) 41 (3.6)
Tyagi et al., 2021 [32]	RSC	- RAPN (201) Hilar (48) Nonhilar (153)	4.7 ^a^ 3.7 ^a^	7.9 ^a^ 7.8 ^a^	162.4 ^a^ 144.1 ^a^	201.8 ^b^ 150.6 ^b^	29.0 ^a^ 24.4 ^a^	3 (6.2) 1 (0.6)	38 ^b^	NR	1 (2) 0 (0)	NR
Hinata et al., 2021 [39]	PMC	- RAPN (105) - LPN	3.2 ^a^	8.7 ^a^	146 ^a^	138 ^a^	20.2 ^a^		24 ^a^	−9.6 ^a^	NR	NR
Chen et al., 2022 [38]	RSC	- RAPN (52) - LPN (64)	4.3 ^a^ 4.1 ^a^	NR	130 ^a^ 126.6 ^a^	100 ^a^ 150 ^a^	20.3 ^a^ 24.5 ^a^	0 (0) 0 (0)	6 ^a^ 6 ^a^	−9.2 ^a^ −13.0 ^a^	1 (1.9) 2 (3)	NR
Ferriero et al., 2022 [42]	ND	- RAPN Off-clamp (10)	3.0 ^b^	10 ^b^	85 ^b^	150 ^b^	NR	0 (0)	27 ^b^	0	0 (0)	1 (5)
Zhang et al., 2022 [41]	RSC	- RAPN (8)	4.2 ^a^	9.5 ^b^	144 ^a^	86 ^a^	27.9 ^a^	0 (0)	NR	NR	0 (0)	NR

CT, computer tomography; EBL, estimated blood loss; eGFR, estimated glomerular filtration rate; LPN, laparoscopic partial nephrectomy; ND, national database; NR, not reported. OPN, open partial nephrectomy; OT, operative time; PMC, prospective multicenter; PSM, positive surgical margins; R.E.N.A.L., (R)adius (tumor size as maximal diameter), (E)xophytic/endophytic properties of the tumor, (N)earness of the tumor’s deepest portion to the collecting system or sinus, (A)nterior (a)/posterior (p) descriptor and the (L)ocation relative to the polar line; RAPN, robot-assisted partial nephrectomy; RMC, retrospective multicenter; RSC, retrospective single center; WIT, warm ischemia time; ^a^ mean. ^b^ median.

## Supplementary Materials

The following supporting information can be downloaded at: https://www.mdpi.com/article/10.3390/cancers16040693/s1, Table S1: Limitations of the included studies.
