# Supplementary material for: Outcomes and Techniques of Robotic-Assisted Partial Nephrectomy (RAPN) for Renal Hilar Masses: A Comprehensive Systematic Review
Source: Cancers (Basel). 2024 Feb 6;16(4):693. doi: 10.3390/cancers16040693 (PMC10886610; doi:10.3390/cancers16040693)
Supplement: Supplementary file 1 [file cancers-16-00693-s001.zip › cancers-2818013-supplementary.pdf]

Supp. Table S1. Limitations of the included studies

Reference	Retrisoective Study design	Multicenter Study design	National Database	Prosective Study Design	Simple size, *n*	Lack* of functional outcome	Lack* of recurrence outcome	Lack* of Follow-up data
Dulabon et al. 2010	x	x			446	X	x	x
Khalifeh et al. 2012	x				15		x	
Eyraud et al. 2013	x				364	x	x	
Miyake et al. 2014	x				31			x
Lu et al. 2018	x				200		x	
Gao et al. 2020	x				286	x		
Sunaryo et al. 2020	x	x			1730		x	x
Mellouki et al. 2020	x		x		1359	x		x
Tyagi et al. 2021	x				201	x	x	
Hinata et al. 2021		x		x	105		x	
Chen et al. 2022	x				116		x	
Ferriero et al. 2022	x		x		10			
Zhang et al. 2022	x				8	x	x	x

* lack / unreliable / non-granular data
